# Molecular Characterization of High Molecular Weight Polyesters by Matrix-Assisted Laser Desorption/Ionization High-Resolution Time-of-Flight Mass Spectrometry Combined with On-plate Alkaline Degradation and Mass Defect Analysis

**DOI:** 10.1007/s13361-018-2092-x

**Published:** 2018-11-08

**Authors:** Sayaka Nakamura, Thierry Fouquet, Hiroaki Sato

**Affiliations:** Research Institute for Sustainable Chemistry, National Institute of Advanced Industrial Science and Technology (AIST), Tsukuba, Ibaraki 305-8565 Japan

**Keywords:** Alkaline degradation, On-plate sample preparation, High molecular weight polyesters, MALDI TOF MS, High-resolution mass spectrometry, Kendrick mass defect analysis

## Abstract

Matrix-assisted laser desorption ionization high-resolution time-of-flight mass spectrometry (MALDI HR TOF MS) is a powerful tool for the molecular characterization of industrial polymers. However, accurate mass determination and resolution of isobaric ions are possible for oligomer samples only typically below *m/z* 3000. To cut long polymer chains into oligomers suitable for high-resolution mass spectrometry, we propose a simple “on-plate” alkaline degradation of polyesters as a sample pretreatment technique prior to the MALDI TOF MS measurement. This pretreatment can be performed on a MALDI target using a small amount of sample (μg or less) and 1 μL of alkaline reagent by simple pipetting. Informative mass spectra in the oligomeric mass range are successfully recorded but complicated by the variation of end-groups and the copolymeric composition of the degradation products. Data processing is assisted by a series of advanced Kendrick mass defect (KMD) analyses recently proposed by the authors to plot visually understandable two-dimensional maps. On-plate degradation pretreatment, high-resolution MALDI TOF MS measurements, and advanced KMD analyses are innovatively combined for the compositional characterization of bacterial poly(3-hydroxybutyric acid*-co-*3-hydroxyvaleric acid) and industrial poly(ethylene terephthalate) samples.

**Electronic supplementary material:**

The online version of this article (10.1007/s13361-018-2092-x) contains supplementary material, which is available to authorized users.

**Graphical Abstract:**
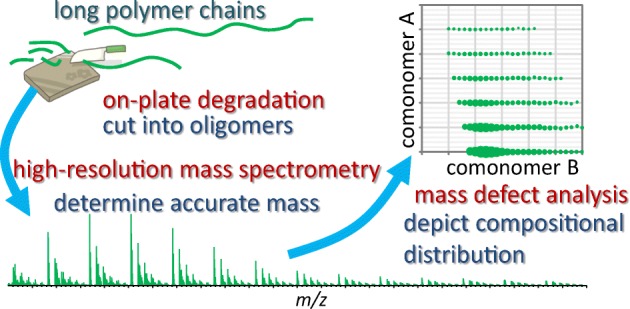
ᅟ

## Introduction

Matrix-assisted laser desorption ionization time-of-flight mass spectrometry (MALDI TOF MS) is an effective method for the characterization of polymers revealing their molecular weight distribution and the nature of their end-groups [1]. Thanks to a remarkable progress in analytical instrumentation last decade, a cutting-edge mass spectrometer with a unique 17-m-long spiral-shaped TOF analyzer [2, 3] makes possible the discrimination of isobaric ions as a valuable feature for the characterization of polymers [4–6]. However, high-resolution/high-accuracy mass measurements are possible for oligomer samples only with a mass range no greater than *m/z* 3000. The oligomeric fraction (if existing) may nevertheless not reflect the whole polymer sample in terms of end-groups and copolymeric composition, casting doubts on the capability of MS for the characterization of industrial high molecular weight samples.

To overcome this issue, Arakawa and co-workers [7–9] introduced the ultrasonic degradation of polymers prior to their analysis by MALDI TOF MS. They reported that various end-groups were generated by sonication, while sufficient chain shortening required a long treatment time—typically several hours—due to low efficiency of the ultrasonic degradation. Owing to these limitations, the ultrasonic degradation has not been used in practice for the sample preparation so far.

Pyrolysis is more commonly used as a mean of generating low molecular weight products from synthetic polymers and natural products. Pyrolysis gas chromatography (Py-GC) is the preferred technique for the molecular and structural characterization of high molecular weight (as well as cross-linked) polymers [10]. Flash pyrolysis at high temperature exceeding 400 °C is suitable to obtain monomeric products while information about the sequence in the original polymer is lost. Alternatively, partial pyrolysis at lower temperatures might be an effective approach to produce oligomeric products. In the case of polyesters, their pyrolysis is well known to form oligomers carrying carboxyl and olefin end-groups upon a β-hydrogen elimination. Based on this reaction, partial pyrolysis has been applied for the characterization of bacterial copolyesters in combination with fast atom bombardment (FAB)-MS [11], electrospray ionization (ESI)-MS [12], or MALDI TOF MS [13]. In these reports, the sample was heated until it suffered a 20% weight loss as followed by thermogravimetric (TG) measurement. As such, it required a few milligrams of sample for accuracy reasons. Reproducing the experiments, we found that the temperature control is difficult and that a dedicated rapid cooling apparatus is required to immediately quench the degradation reaction at 20 wt% weight loss.

Acid- or base-catalyzed transesterification would be another approach to softly decompose high molecular weight polyesters into oligomers. Prior to MALDI TOF MS measurements, hydrochloride and sodium methoxide in methanol were used as the reagents for the partial transesterification of poly(3-hydroxybutyric acid*-co-*3-hydroxyvaleric acid) (P(3HB*-co-3*HV)) [14] and poly(β-hydroxyalkanoate)s (PHAs) [15], respectively. However, these reactions were carried out at flask scale. It may thus require a trial-and-error optimization of the reaction conditions to produce the degradation products having suitable molecular weights for a high-resolution mass spectral characterization.

In this paper, we propose a simple pretreatment method for MALDI TOF MS measurements of high molecular weight polyesters as a modification of the partial alkaline transesterification previously reported [15]. In essence, the main feature of our method is that the pretreatment of a polymer sample—either a thin layer drop-casted from a solution or a film—can be performed on a MALDI target plate within a few minutes by pipetting an alkaline solution. This idea originated from the industrial processing of polyester fibers. In the field of textile processing, surface modification by alkaline treatments has been used to improve the properties of polyester fibers as a finishing technique [16]. In tissue engineering, the cell affinity of polyester-based materials has also been improved via their surface modification by alkaline hydrolysis treatment [17–20]. In both cases, molecular weight reduction of polyesters has been found to occur at the surface layer only. This phenomenon would be convenient for the characterization of high molecular weight polyesters by MALDI TOF MS. Despite a wide range of molecular weights from oligomeric degradation products to intact polymer chains, the surface layer of a partially degraded sample would indeed concentrate the oligomers without any optimization of the reaction conditions.

A last difficulty remains considering that the scission of ester bond by transesterification produces several different types of end-groups. For the characterization of such complex blends, the authors have proposed a new strategy combining the high-resolution MALDI TOF MS measurements with a Kendrick mass defect (KMD) analysis [6, 21]. A KMD analysis [22] is a data processing tool for the rapid visualization of complex mass spectra traditionally used for petroleomics [23, 24] but recently extended to the field of polymer analysis [6, 21, 25–28]. The two-dimensional plot generated by a KMD analysis displays a relationship between the molecular weight (or degree of polymerization; *x*-axis) and differences in elemental composition reflecting differences in chemical structures (variation of end-groups, comonomer distribution, degree of modification, oxidation, etc.; *y*-axis). More recently, our research group has aggressively proposed a series of advanced KMD analyses for the compositional characterization of copolymers by introducing, refining, or extending the concepts of “fractional base unit” [29–31] and “referenced KMD” [32, 33].

In this paper, a simple alkaline degradation on the MALDI target plate (termed “on-plate degradation”) is first investigated through the MALDI TOF MS analysis of a commercial polyester. Isotopic labeling (reagents and solvent) is used to confirm the degradation scheme. The compositional characterization of high molecular weight bacterially produced copolyester and industrial polyester is then carried out to demonstrate that combining the on-plate degradation and the advanced KMD analysis pushes the boundaries of high-resolution MALDI TOF MS.

## Experimental

### Chemicals and Materials

**Scheme 1:**
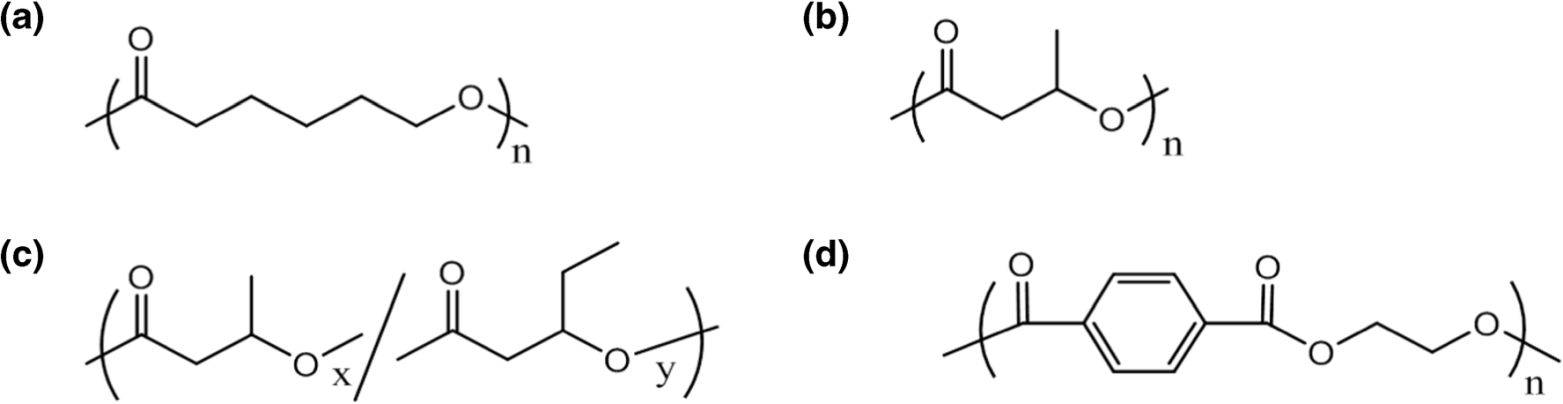
Structures of the four high molecular weight polyesters: (**a**) PCL, (**b**) P3HB, (**c**) P(3HB-*co*-3HV) ,and (**d**) PET

Poly(ε-caprolactone) (PCL, average molecular weight at ca. 10000 g mol^−1^ according to the supplier), poly(3-hydroxybutyric acid) (P3HB, *M*_n_ = 2.6 × 10^5^ g mol^−1^, *M*_w_ = 7.1 × 10^5^ g mol^−1^), and P(3HB*-co-*3 HV) (3 HV content = ca. 12 mol%, *M*_n_ = 1.4 × 10^5^ g mol^−1^, *M*_w_ = 3.5 × 10^5^ g mol^−1^) were all purchased from Sigma-Aldrich (St. Louis, MO, USA). The PCL chains have already been found to be formed of an ethylene glycol core used as initiator with two PCL arms both carrying a hydroxyl group as chain-end [6, 34]. A commercial poly(ethylene terephthalate) (PET) film (Lumirror™ S10, thickness ca. 23 μm, Toray Industry, Tokyo, Japan) and a commercial PET bottle were also used as examples of high molecular weight polyesters. Structures of the polymers are depicted in Scheme 1.

As an alkaline reagent for the on-plate degradation, sodium hydroxide (NaOH, reagent grade, Wako pure chemicals, Osaka, Japan) was dissolved in methanol (CH_3_OH, infinity pure grade, Wako) or methanol-*d*_4_ (99.8 atom%, Wako) at ca. 10 mg mL^−1^. A 28% solution of sodium methoxide (CH_3_ONa) in methanol (Wako) was diluted to ca. 10 mg mL^−1^ in methanol. All deuterated alkaline reagents of sodium deuteroxide (NaOD) in methanol-*d*_4_ were prepared as follows: ca. 10 μL of a 40% solution of NaOD in deuterium oxide (D_2_O, 99.5 atom%) purchased from Alfa Aesar (Lancashire, UK) was vacuum-dried to remove D_2_O and ca. 40 μL of methanol-*d*_4_ was added to form a methanolic solution at ca. 10 mg mL^−1^. Distilled water or D_2_O (100 atom%, Wako) was used to wash the excess amount of the alkaline reagent on the MALDI target.

Tetrahydrofuran (THF, reagent grade), 1,1,1,3,3,3-hexafluoro-2-propanol (HFIP), and chloroform (HPLC grade) were from Wako and used as solvent for the polymer samples and/or as mobile phase for size-exclusion chromatography (SEC). 2,4,6-Trihydroxyacetophenone (THAP, ultrapure grade, Protea Biosciences, West Virginia, USA) was used as a MALDI matrix and dissolved in THF at ca. 20 mg mL^−1^.

### SEC Measurement and Fractionation

As a fundamental study, the SEC fractionation was carried out to confirm the degradation products of high molecular weight polyesters. Average molecular weights of P3HB and P(3HB*-co-*3HV) were determined by a build-up LC system (Tosoh, Tokyo, Japan) composed of an AS-8020 auto sampler, a CCPS pump, an SD-8022 degasser, a CO8020 column oven, and a RI-8020 refractive index detector (RID). Ten microliters of the sample solution at 2 mg mL^−1^ in HFIP were injected and analyzed using two TSKgel super HM-H columns (6.0 mm × 150 mm) connected in series following an HM-H guard column with HFIP containing 0.5 mM sodium trifluoroacetate as the mobile phase (0.3 mL/min). A calibration curve was made using a kit of poly(methyl methacrylate) standards (PMMA M-75 kit, Shodex, Tokyo, Japan).

A high molecular weight fraction of PCL was recovered from a SEC elution of the commercial PCL using a HLC8220 system (Tosoh) equipped with a RID. The sample solution (200 μL at 2 mg mL^−1^ in CHCl_3_) were fractionated using two TSKgel multipore HXL-M columns (7.8 mm × 300 mm) connected in series following a multipore Hxl guard column with CHCl_3_ as the mobile phase (1 mL min^−1^). Aliquots of 0.5 mL (30 s of elution) were collected in vials directly after the RID. The concentration of the chosen fraction could be estimated as 0.08 mg mL^−1^ based on the RID signal intensity. The number-average molecular weight (*M*_n_) of the fractionated sample was about 13,800 g mol^−1^ (Supporting Information Figure SI-1, MALDI TOF mass spectrum in linear mode) and no peaks were observed in the MALDI spiral TOF mass spectrum under *m/z* 10,000.

### On-plate Degradation Procedure

All samples but PETs were dissolved in THF at ca. 1 mg mL^−1^. About 1 μL of each sample solution was drop-casted on a disposable MALDI target plate (non-focus, non-hydrophobic grade, Hudson Surface Technology, New Jersey, USA) to form a polymer thin layer. The PET samples were cut by a micro puncher (inner diameter 0.75 mm, Frontier Lab., Koriyama, Japan) and directly put on the MALDI target plate. Next, ca. 1 μL of HFIP was added on each PET pellet to form a polymer thin layer around it. Then, ca. 1 μL of the alkaline reagent was deposited over the sample layer and left to air-dry for about 5 min until methanol fully evaporated. The excess amount of the alkaline reagent was washed with ca. 5 μL of distilled water or D_2_O by pipetting (discharge/suction repeated three times) following the same procedure as an on-plate desalting for MALDI sample preparation [35]. After drying, ca. 1 μL of the matrix solution was applied on the sample spot.

### MALDI TOF MS Measurements and Data Processing

MALDI TOF MS measurements were performed using a JMS-S3000 spiralTOF™ (JEOL, Akishima, Japan). This apparatus is equipped with an Nd: YFL laser (λ = 349 nm) and a spiralTOF™ analyzer with a spiral ion trajectory corresponding to a flight length of approximately 17 m. The apparatus parameters were set to maintain ∆M < ca. 0.03 Da at FWHM over the range of *m/z* 800–3000. Mass calibration was made with a PMMA standard (peak-top molecular weight, *M*_p_ = 1310) purchased from Polymer Laboratories (Shropshire, UK).

Mass spectra were processed and exported using MS Tornado Analysis software (JEOL). KMD analyses of the exported MS data were performed using msRepeatFinder software ver. 2.1.0 (JEOL). The referenced KMD analysis to depict the compositional distribution of P(3HB-*co*-3HV) was eventually carried out using Excel and a home-made set of macros (VBA coding).

## Results and Discussion

### Fundamental Study of the On-plate Degradation

**Figure 1:**
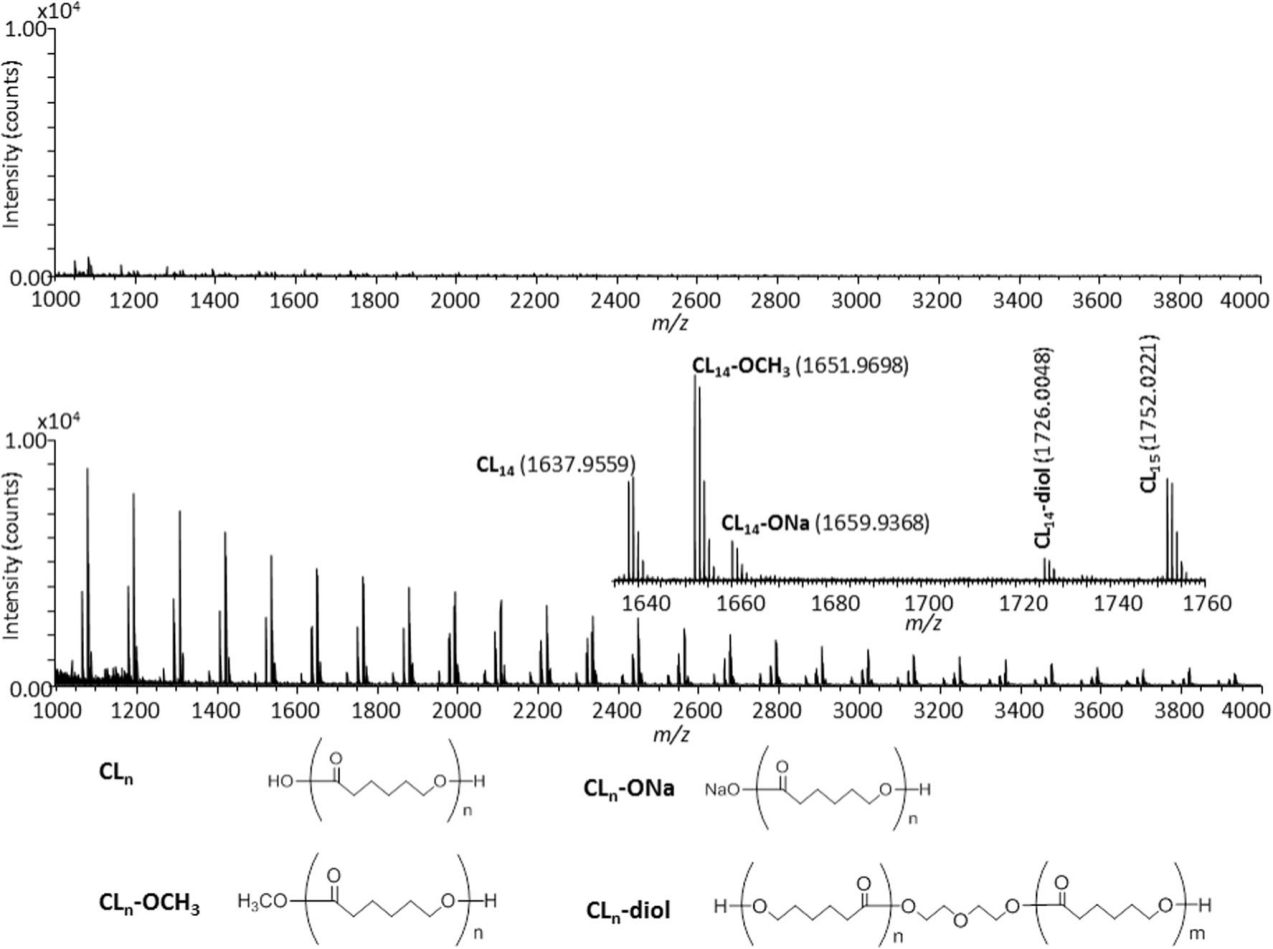
Mass spectra of the fractionated PCL following the on-plate degradation procedure using CH_3_ONa in methanol (**a**) before and (**b**) after washing with distilled water

The experimental conditions of the on-plate degradation were optimized using the high molecular weight PCL sample (*M*_n_ = 13,800 g mol^−1^) having no oligomeric components, whose fractionation procedure is described in the Supporting Information. As a first trial, the fractionated PCL deposited on the target plate (ca. 80 ng/spot) was treated with ca. 1 μL of CH_3_ONa in methanol used as the alkaline reagent. Figure 1a shows the mass spectrum of the fractionated PCL after its on-plate degradation. A few oligomeric degradation products are barely observed in the background. Such a low peak intensity suggests that a strong ion suppression effect occurs caused by an excess amount of the alkaline reagent as also suspected by the presence of a white deposit on the sample spot after the evaporation of methanol. To remove the excess of alkaline reagent, an additional washing with distilled water was carried out as for an on-plate desalting [35]. The mass spectrum recorded after washing by simple pipetting is depicted in Figure 1b and clearly displays oligomeric peaks with strong intensities (*y*-axis of two mass spectra are set at the same intensity range to compare absolute abundances). Four peak series of degradation products are clearly observed. The peak series noted **CL**_**n**_ is identified as *n*-mers of PCL with carboxyl and hydroxyl end-groups. The peak series noted **CL**_**n**_**-OCH**_**3**_ and **CL**_**n**_**-ONa** are the methyl ester and the sodium salt of carboxylic acid of **CL**_**n**_. The peak series noted **CL**_**n**_**-diol** corresponds to a dihydroxyl-ended PCL containing a diethylene glycol unit in the main chain—a key product to ensure the high molecular weight PCL also contains the diethylene glycol unit [6, 34]. The chemical structures of the four oligomeric series are depicted below the mass spectrum.

**Figure 2:**
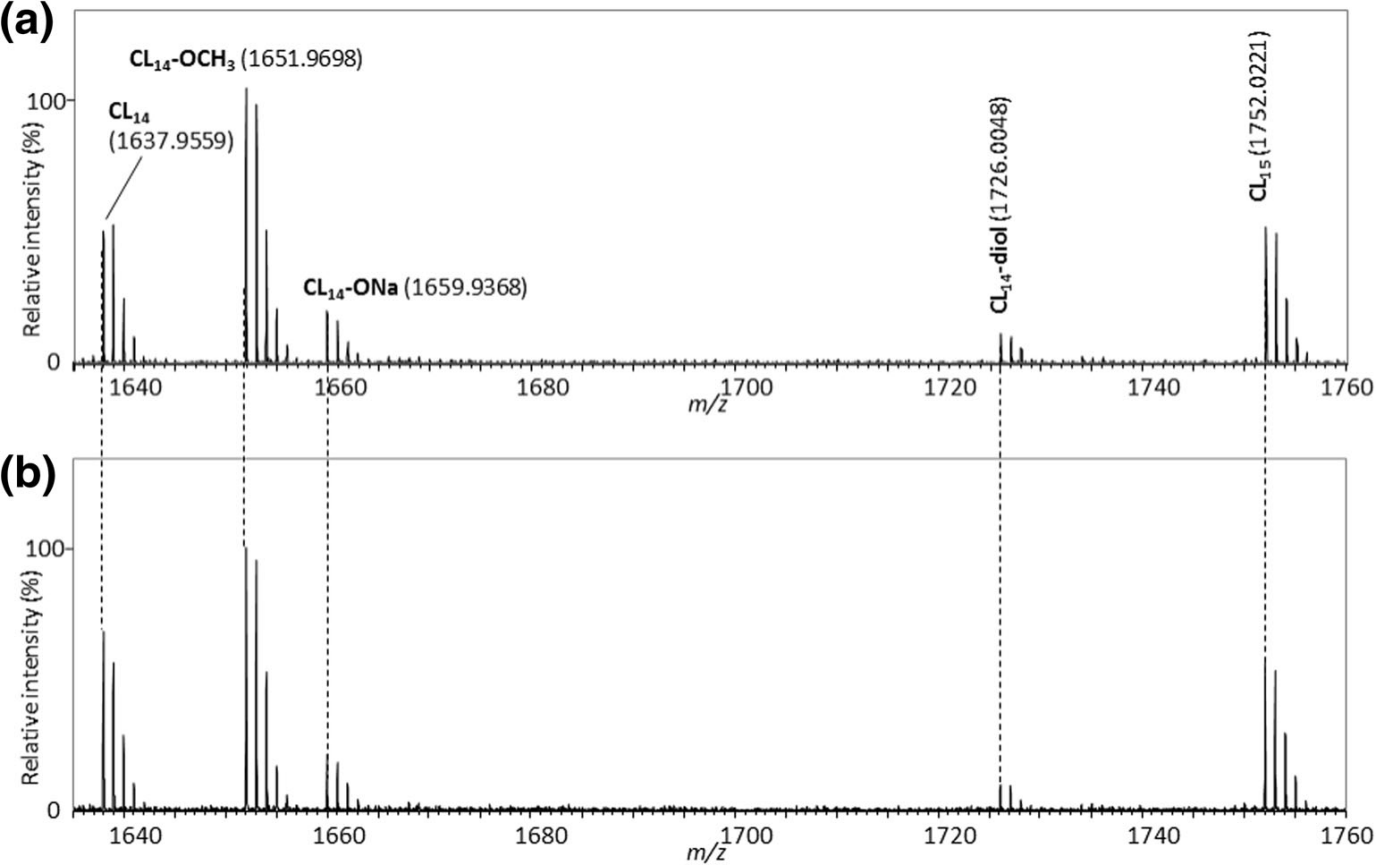
Comparison of mass spectra of the fractionated PCL processed by the on-plate degradation using CH_3_ONa in methanol followed by washing with (**a**) H_2_O and (**b**) D_2_O

As demonstrated above, the on-plate degradation procedure necessarily requires the removal of excess alkaline reagent using distilled water. As the possibility of hydrolysis to occur during the washing procedure cannot be dismissed, a comparative experiment was conducted using D_2_O instead of distilled water to highlight unwanted hydrolysis products via isotopic shift. Figure 2 shows the mass spectra of the fractionated PCL after the on-plate degradation using CH_3_ONa in methanol and washing with H_2_O (Figure 2a, cf. inset in Figure 1b) and D_2_O (Figure 2b). The absence of any peak shift (–OH to –OD, + 1 Da) ensures that no hydrolysis occurs during the washing step as an unwanted side reaction.

**Figure 3:**
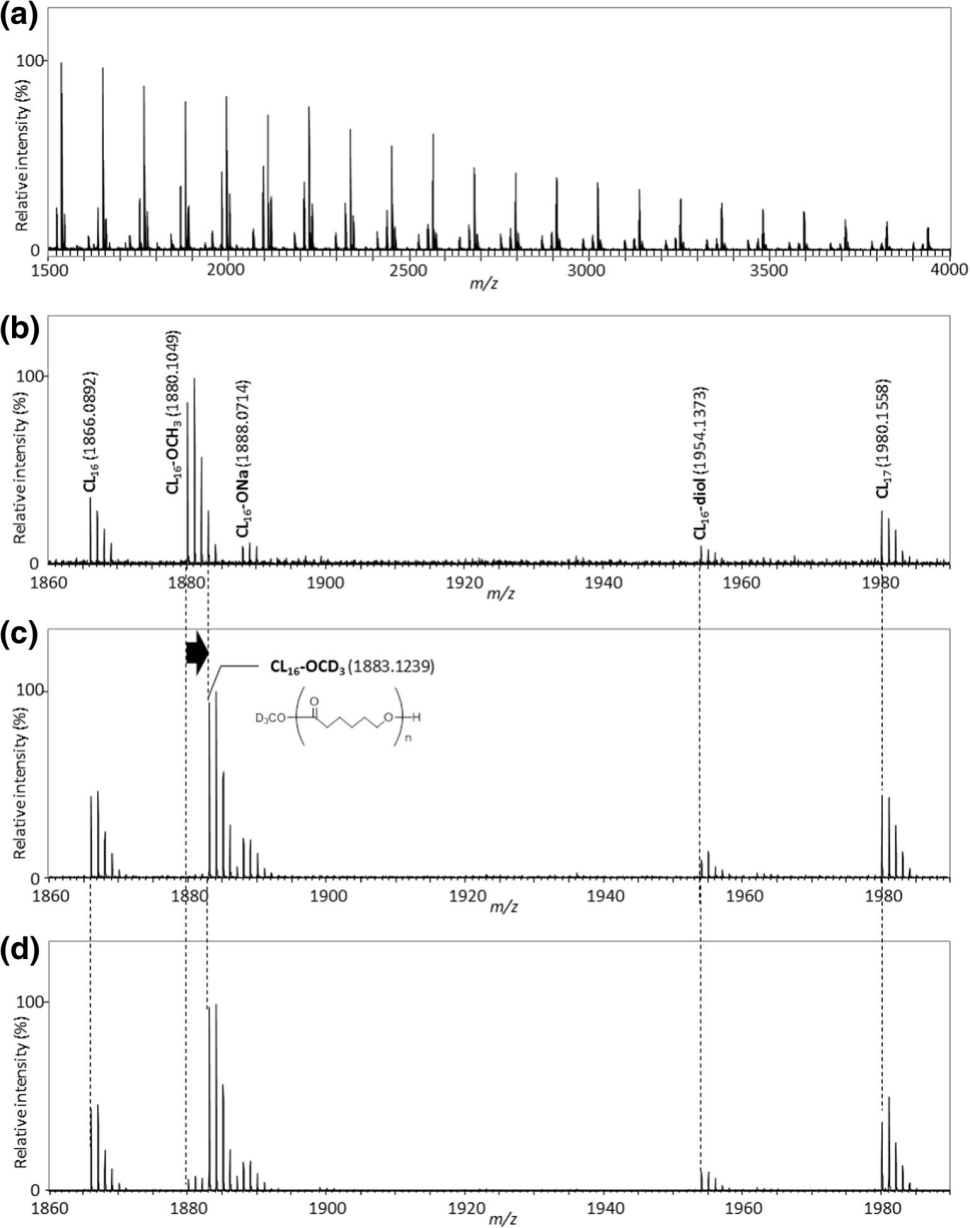
Comparison of the mass spectra of the fractionated PCL after the on-plate degradation using isotope-labeled reagents. (**a**) Wide band mass spectrum of the fractionated PCL processed by NaOH in methanol followed by washing with water. (**b**)**–**(**d**) are the expanded mass spectra. The combinations of degradation reagent and washing solvent are (**a**) and (**b**) NaOH in methanol and distilled water, (**c**) NaOH in methanol-*d*_4_ and distilled water, and (**d**) NaOD in methanol-*d*_4_ and D_2_O

The main products formed upon the on-plate degradation of the fractionated PCL are PCL oligomers bearing carboxyl and hydroxyl end-groups together with oligomers having methyl ester terminations. To discuss the formation mechanisms of these degradation products, deuterated solvents and alkaline reagents such as methanol-*d*_4_ and D_2_O were used separately or all together. In order to limit the origin of methyl ester terminal to the sole PCL backbone, NaOH and its deuterated NaOD counterpart were used as the alkaline reagents instead of CH_3_ONa. Figure 3 compares the mass spectra of the fractionated PCL after its degradation and washing using NaOH in methanol and distilled water (no isotopic labeling), NaOH in methanol-*d*_4_ and distilled water (labeling of solvents), and NaOD in methanol-*d*_4_ and D_2_O (full labeling). NaOH in methanol has evidently the same ability to degrade the PCL sample as CH_3_ONa (Figures 1 and 2) since oligomeric PCL series are detected over the whole mass range (Figure 3a) and discrete degradation products are identical (Figure 3b, no isotopic labeling). When methanol-*d*_4_ was used as the solvent for NaOH (washing with H_2_O), the peak series **CL**_**n**_**-OCH**_**3**_ with the methyl ester end-group almost disappeared while a new peak series **CL**_**n**_**-OCD**_**3**_ appeared instead (Figure 3c). An unexpected + 3 Da peak shift was observed for this ion series in lieu of the expected + 4 Da shift (original end-groups without labeling: H_3_CO, H; deuterium-labeled end-groups: D_3_CO, D; + 4 Da), indicating that the deuterium replacement occurred only at the methyl ester termination while the terminal hydroxyl group was left untouched. The three other peak series **CL**_**n**_, **CL**_**n**_**-ONa**, and **CL**_**n**_**-diol** did not show any peak shifts, suggesting again that deuterium was hardly introduced to the hydroxyl and carboxyl end-groups. Finally, all chemicals (alkaline reagent and solvents) were changed to their deuterated counterparts, i.e., NaOD in methanol-*d*_4_ as the alkaline reagent and D_2_O as the washing solvent. Even in this case, no deuterium replacement did occur either at the carboxyl or at the hydroxyl end-groups (Figure 3d).

From these results, we can infer that the origin of hydrogen atoms in the carboxyl and hydroxyl end-groups would be the PCL molecules themselves. The alkaline degradation of polyesters has been well investigated in the literature. The methoxide anion in the reaction solvent nucleophilically attacks the carbonyl of the ester bond producing methyl esters. If the alkaline degradation using deuterium-labeled reagents is performed completely to yield monomeric products, the hydrogen atoms of their hydroxyl groups should all be replaced with deuterium. However, the alkaline degradation reaction by the on-plate degradation procedure is surely not complete as it occurs at the surface layer only, leaving a considerable amount of intact carboxyl end-groups near the reaction sites. So, the protons released from the carboxyl end-groups would connect with alkoxide anion and form hydroxyl end-groups free of deuterium despite using deuterium-labeled reagents.

### Application for the Compositional Analysis of High Molecular Weight Copolymers

Amid growing interest in environmental issues, PHAs produced by bacteria are seen as one of the most promising candidates for renewable materials. Since the chemical information such as the type of monomers and the copolymeric composition of PHAs generally reflects the carbon source fed to the bioreactor [36], the analysis of PHAs is critical from the view point of quality control. However, the high molecular weight of bacterial PHAs typically over hundreds of thousands Da makes the mass analysis of intact PHAs difficult especially for the evaluation of co-monomeric content [14]. In this study, the capability of the on-plate degradation combined with high-resolution MALDI TOF MS was demonstrated for the thorough characterization of high molecular weight PHAs.

**Figure 4:**
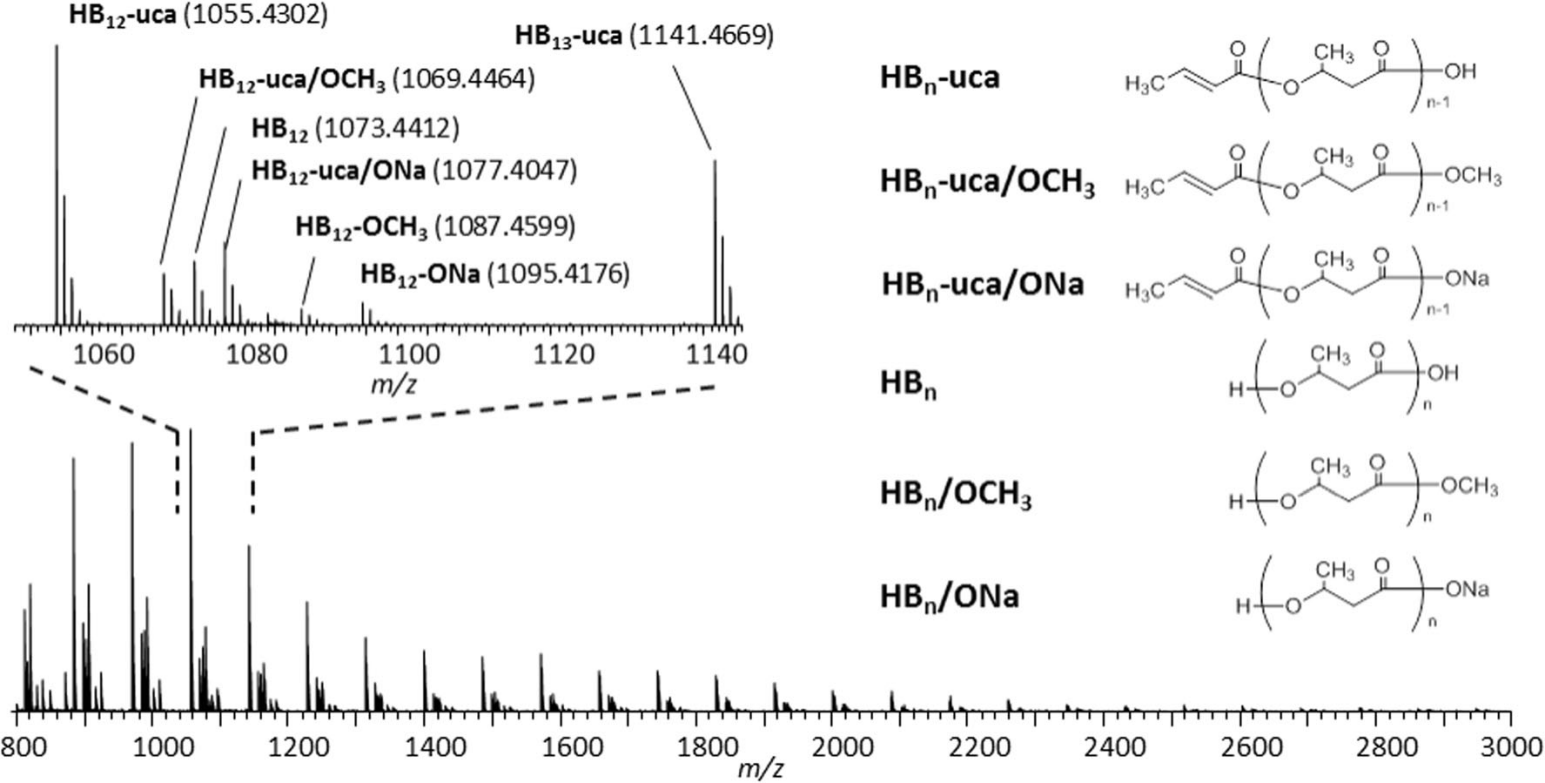
Mass spectrum of a high molecular weight P3HB following its on-plate degradation using NaOH in methanol

Prior to the analysis of a P(3HB-co-3HV) copolymer, a first demonstration was performed for the case of P3HB homopolymer to confirm the formation scheme of end-groups. Figure 4 shows the mass spectrum of P3HB processed by the on-plate degradation with NaOH in methanol. At least six peak series spaced by 86 Da corresponding to the expected repeating unit are observed (3-hydroxybutyric acid C_4_H_8_O_3_, associated repeating unit via polymerization: C_4_H_6_O_2_, Scheme 1). Peak assignments were made on the basis of accurate mass measurements and by referring to the literature [15]. The main degradation product series is proposed to carry (CH_3_CH=CHCO–, –OH) as end-groups corresponding to an unsaturated crotonic acid moiety (CH_3_CH=CHCO_2_H). Although no unsaturated end-group was produced in the case of PCL degradation, this assignment remains reasonable since unsaturated end-group is known to be specifically formed by alkali hydrolysis of P3HB [37].

The on-plate degradation as the pretreatment for the high-resolution MALDI TOF MS measurements was further applied for the compositional characterization of P(3HB-*co-*3HV) copolymer. An isomeric issue arises here as the mass difference of 14 Da (CH_2_) between 3HB and 3HV co-monomeric units is the same as the mass variation caused by the esterification of the carboxyl group. To overcome this issue, the deuterium-labeled methanol-*d*_4_ was used as the degradation solvent to induce a mass variation + 17 Da (+CD_3_ –H, + 3 Da shift) for the methyl esters as described in the previous section.

**Figure 5:**
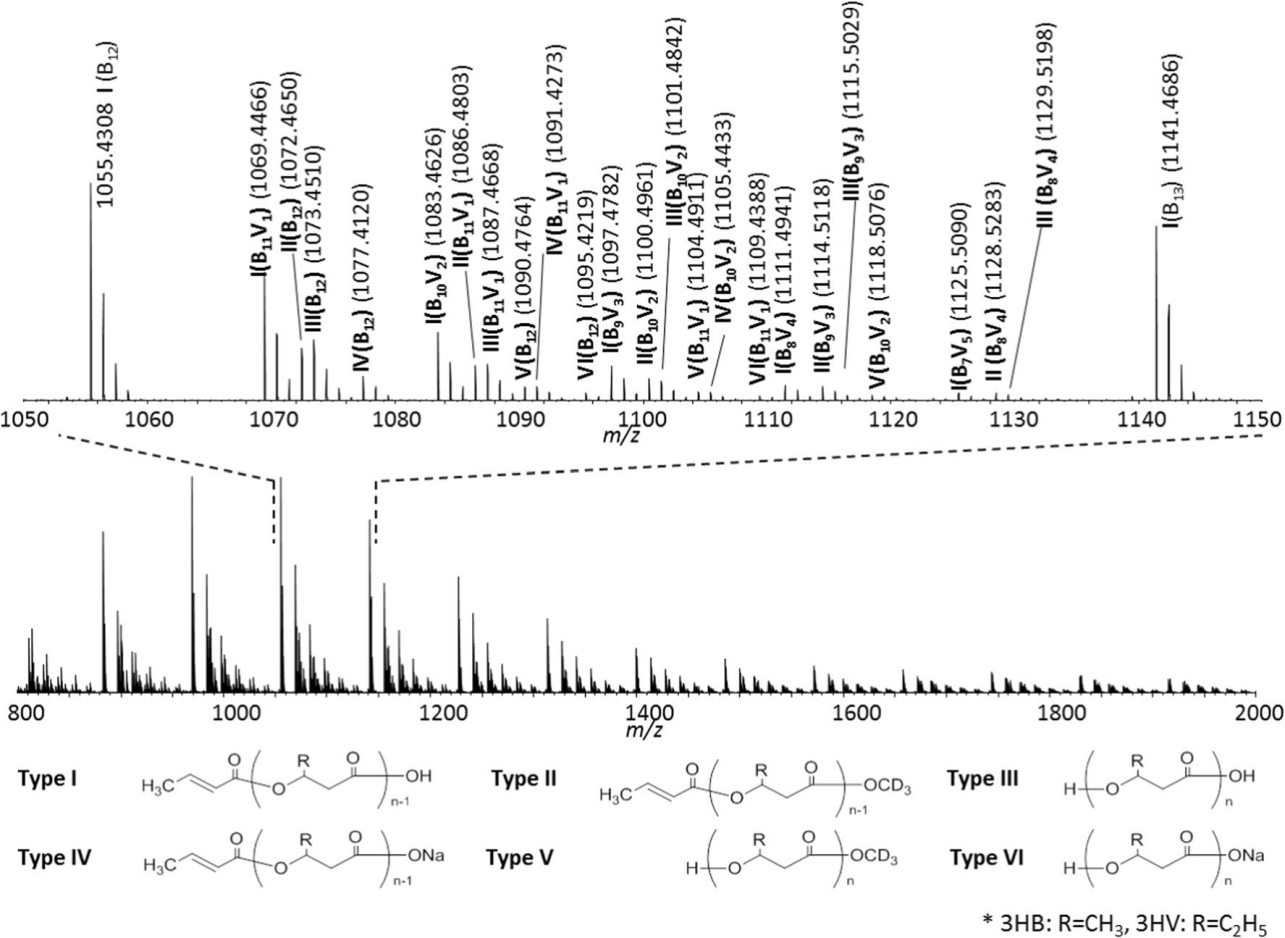
Mass spectrum of P(3HB*-co-*3HV) processed by the on-plate degradation using NaOH in methanol-*d*_4_. Peak assignments are indicated in the expanded mass spectrum, where the roman figure indicates the type of end-group combination shown below the spectrum and (B_n_V_m_) corresponds to the comonomer composition of n units of 3HB and m units of 3HV

Figure 5 shows the mass spectrum of high molecular weight P(3HB*-co-*3HV) processed by the on-plate degradation using NaOH in methanol-*d*_4_. The six types of end-group combinations (type **I** to type **VI**) predicted from the degradation of P3HB homopolymer (Figure 4) are detected in the mass spectrum. A multiplication of peaks nevertheless arises from the distribution of copolymeric composition (varying number of 3HB and 3HV units within a copolymeric backbone) and the end-group combinations mentioned above. Peak assignments are exemplified in the expanded view of Figure 5 with the types of end-group combination and their copolymer compositions. Of note, the signals of the methyl esters (type **II**) are successfully differentiated from type **I** by using methanol-*d4*.

The compositional characterization of copolymers requires a full assignment for a huge number of observed peaks which remains a tedious task. Even if an automatic peak picking using appropriate programs would help for this purpose, a perfect mass calibration is still needed over the whole mass range. Otherwise, inappropriate single setting for the mass tolerance often leads to incorrect peak picking especially for complicated mass spectra. In order to facilitate the data processing avoiding the risk of the incorrect peak assignment, we have proposed a series of advanced KMD analysis techniques [29–33]. The final goal of this section is to display a degree of polymerization (DP) plot [32] as an instant graphical visualization of the comonomer distribution of P(3HB*-co-*3HV).

A first step consisted in extracting a specific series of products with a comonomer distribution (variation of 3HB and 3HV content) but the same end-groups from the whole mass spectral data by means of a resolution-enhanced KMD analysis [29–31]. This approach is an improved version of a regular KMD analysis [22–24] introducing the concept of a fractional base unit [29]. The accurate *m/z* values of ions in the IUPAC scale are converted to Kendrick mass (KM_*r/X*_) according to

1 $$ {\mathrm{KM}}_{r/X}=m/z\bullet \frac{\mathrm{round}\left(R/X\right)}{R/X} $$

where *R* is the exact IUPAC mass of the repeating unit *r* of a given polymer, round(*x*) is the nearest integer function following the round half up rule, and *X* is any integer (*X* = 1 for the regular KMD analysis), respectively. Two important parameters known as the nominal KM_*r/X*_ (noted NKM_*r/X*_) and the Kendrick mass defect (KMD_*r/X*_) can be calculated according to

2 $$ {\mathrm{NKM}}_{r/X}=\mathrm{round}\left({\mathrm{KM}}_{r/X}\right) $$

3 $$ {\mathrm{KM}\mathrm{D}}_{r/X}={\mathrm{NKM}}_{r/X}-{\mathrm{KM}}_{r/X} $$

Usually, data triplets (NKM_*r/X*_, KMD_*r/X*_, abundance) are plotted in a two-dimensional “bubble chart” with NKM_*r/X*_ on the *x*-axis and KMD_*r/X*_ on the *y*-axis to produce a KMD plot. However, in this report, nominal mass of *m/z* was set for the *x*-axis, because NKM_*r/X*_ greatly deviates from nominal mass depending on the value of *X*.

**Figure 6:**
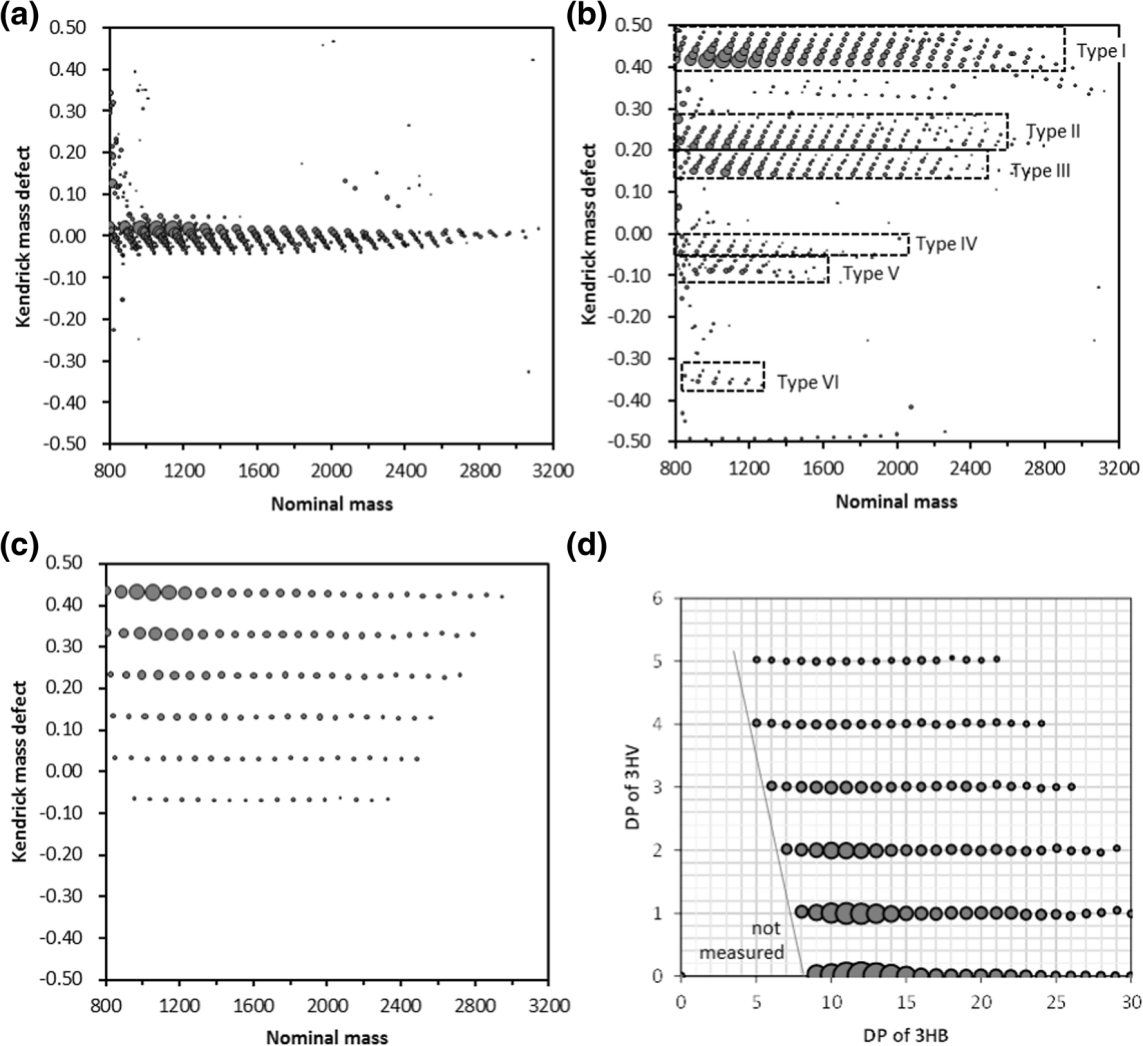
KMD plots and DP plot of P(3HB-co-3HV) processed by the on-plate degradation using NaOH in methanol-*d*_4_. (**a**) Regular KMD plot (repeat unit: C_4_H_6_O_2_, 86.03623), (**b**) resolution-enhanced KMD plot (repeat unit 86.03623, divisor *X* = 92), (**c**) resolution-enhanced KMD plot for the type I cloud extracted from (**b**) (divisor *X* = 62), and (**d**) DP plot (divisor *X* = 62 for 3HB, *X* = 114 for 3HV)

Figure 6 displays the KMD plots from the deisotoped mass spectral data of the degradation products of P(3HB-*co*-3HV) with different KMD data processing methods, using 3HB as the base unit (*r* = 3HB and *R* = 86.0368). The regular KMD plot with *X* = 1 in Eq. (1) is shown in Figure 6a, where the compositional distribution of 3HB units extends in the *x*-axis direction. However, the dispersion in the *y*-axis direction is insufficient to distinguish the compositional distributions of 3HV units and different end-group combinations. Varying the value of the divisor *X* from 57 to 143 according to the recommended range between $\mathrm{round}\left(\frac{2}{3}R\right)\ \mathrm{and}\ \mathrm{round}\left(\frac{5}{3}R\right)$ [30], a highly favorable situation, is reached at *X* = 92 (Figure 6b). In this case, copolymers are mainly separated based on their end-groups (types **I** to **VI**) much more than by their comonomer content. The cluster of type **I** (the main products) was extracted from the whole resolution–enhanced KMD plot using a data extraction function of msRepeatFinder (known as “grouping mode”).

The next step was the rearrangement of the extracted dataset by another resolution-enhanced KMD analysis with an alternative divisor to better expand the comonomer distribution (detailed data processing is described in the Supporting Information). The resolution-enhanced KMD plot for the extracted type **I** is shown in Figure 6c, where *X* = 62 was set for 3HB. The components of type **I** are now mainly separated based on their content in 3HV (one horizontal line per 3HV unit) while points within a given line differ by their content in 3HB.

A last step was the conversion of the resolution-enhanced KMD to the DP plot to replace the abstruse KMD and *m/z* values (*y*-axis and *x*-axis) by the degree of polymerization in 3HV and 3HB, respectively. For this purpose, the concept of the referenced KMD [38] has been modified by the authors for the case of polymer ions as described elsewhere [32]. The KMD value of each component can be converted to the DP of comonomer B (DP(B)) according to

4 $$ \mathrm{DP}(B)=\frac{{\mathrm{KMD}}_{A/X}-\mathrm{reference}\ {\mathrm{KMD}}_{A/X}\left(\mathrm{residual}\ \mathrm{mass}+\mathrm{cation}\ \mathrm{mass}\right)}{{\mathrm{KMD}}_{A/X}(B)} $$

where reference KMD is the mass defect of the partial mass excluding the main chain from the whole ions, i.e., the sum of residual mass and cation. In this study, the mass of cation is set at 22.9892 Da as all ions are sodium adducts. Originally, the residual mass equals to the sum of the masses of end-groups. However, the sum of the masses of the end-groups for the type **I** ions series is equal to the mass of the 3HB repeating unit itself. In this specific case, the residual mass for the type **I** series is 0 and DP of 3HV can be obtained according to a simplified equation

5 $$ \mathrm{DP}\left(3\mathrm{HV}\right)=\frac{{\mathrm{KMD}}_{3\mathrm{HB}/62}-\mathrm{reference}\ {\mathrm{KMD}}_{3\mathrm{HB}/62}(22.9897)}{{\mathrm{KMD}}_{3\mathrm{HB}/62}\left(\mathrm{HV}\right)}=\frac{{\mathrm{KMD}}_{3\mathrm{HB}/62}-0.4333}{-0.0999} $$

where 3HV = 100.0524 in the IUPAC mass. Exchanging 3HV and 3HB and the divisor *X* set to 114 (well-suited divisor for 3HV within the recommended range from 67 to 200, see the Supporting Information) in Eq. (5), DP values of 3HB for every ion from the type **I** series can also be obtained as follows.

6 $$ \mathrm{DP}\left(3\mathrm{HB}\right)=\frac{{\mathrm{KMD}}_{3\mathrm{HV}/114}-\mathrm{reference}\ {\mathrm{KMD}}_{3\mathrm{HV}/114}(22.9897)}{{\mathrm{KMD}}_{3 HV/114}(HB)}=\frac{{\mathrm{KMD}}_{3\mathrm{HV}/114}+0.1947}{0.0304} $$

The DP plot is finally plotted by displaying the triplets (DP(3HB), DP(3HV), abundance) in a two-dimensional bubble chart (Figure 6d). Detailed explanation to produce the DP plot is additionally described in the Supporting Information. It graphically displays the comonomer distribution of the degraded P(3HB-*co*-3HV). The most abundant products are P3HB homopolymeric oligomers (DP(3HV) = 0) while no P3HV homopolymeric oligomers are observed (DP(3HB)_min_ = 5), suggesting that the original high molecular weight copolymer is a random copolymer with high 3HB content. A block architecture would have indeed released P3HV homopolymeric oligomers upon on-plate degradation. Based on the DP plot, the average 3HV content is calculated at 8.9 ± 0.2 mol% for three sample spots in moderate agreement with the 12 mol% content provided by the supplier. Such a rapid compositional characterization of a high molecular weight copolyester out of reach of a direct MALDI TOF MS analysis has been made possible by the successful combination of the on-plate degradation and the advanced KMD analysis.

### Application for the Characterization of PET Film and Bottle Samples

Considering that the partial pyrolysis preparation requires a few mg of sample for the TG pyrolysis [11–13], there is a clear advantage using the on-plate degradation technique which requires ten times lower amount of sample. Accordingly, the next application was proposed with a view to local analysis of industrial materials. Industrial PET film and bottle were subjected for the on-plate degradation as model samples with ca. 14 μg of PET and 1 μL of NaOH methanol solution.

**Figure 7:**
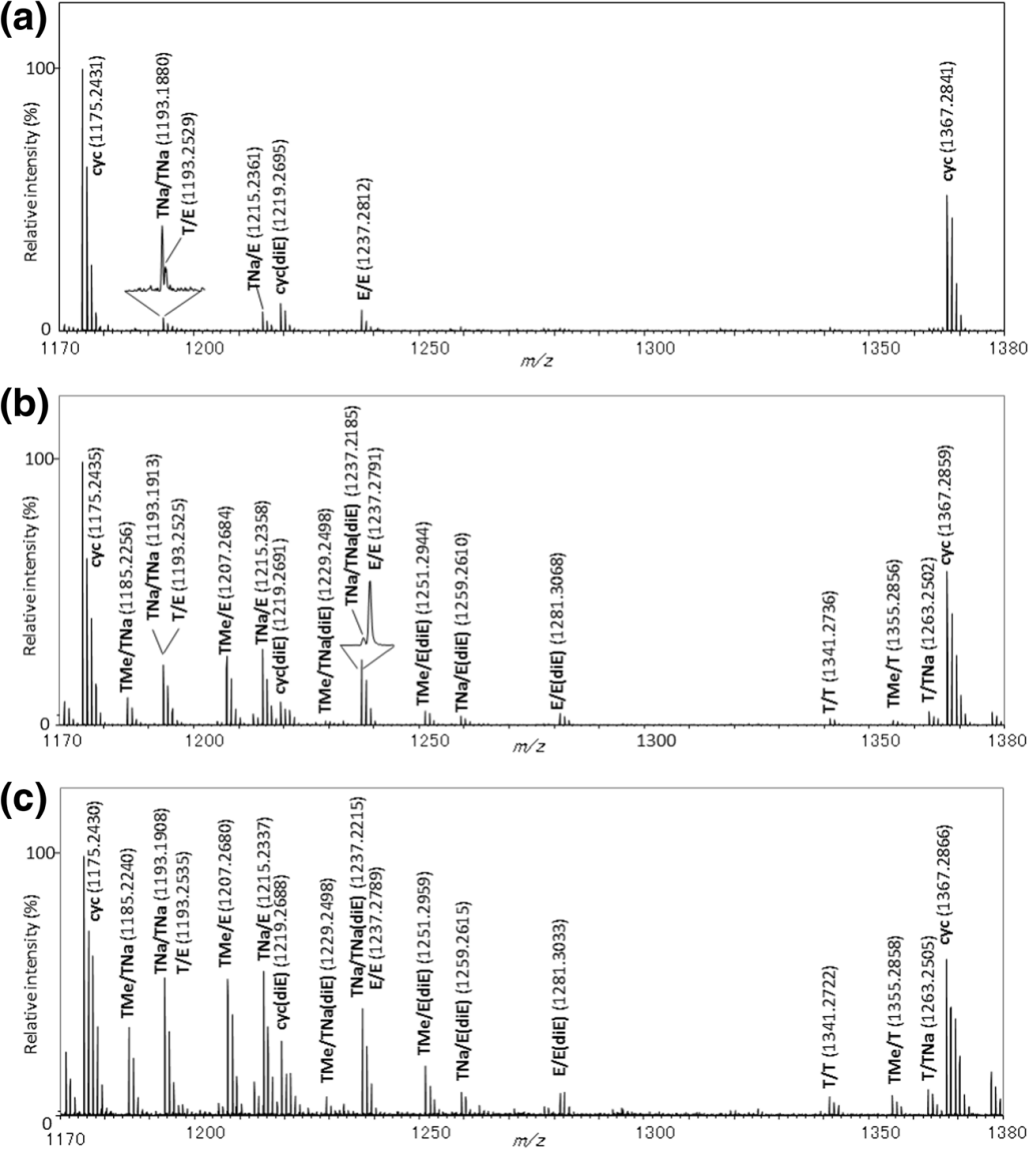
Comparison of expanded mass spectra for **a** pristine PET film, **b** PET film, and **c** PET bottle samples processed by the on-plate degradation. The peak labels and their structures are listed in Table 1

Figure 7 shows the expanded mass spectra from the PET samples with peak assignments and KMD values being summarized in Table 1. Cyclic oligomers (noted **cyc**) at *m/z* 1175.2431 for 6-mer and *m/z* 1367.2841 for 7-mer are mainly detected in the mass spectrum of the pristine PET film (Figure 7a) together with a few other oligomers of low abundance such as a carboxylic acid/hydroxyl ended oligomer (noted **T/E**), its sodium salt (**TNa/E**), or the sodium salt of dicarboxylic acid-ended oligomer (noted **TNa/TNa**). The mass spectra of the on-plate degraded PET film and PET bottle are shown in Figure 7b, c and display very similar fingerprints. In both mass spectra, numerous peaks are detected as a positive result of an effective alkaline degradation. There is no difficulty to identify the products having carboxylic acid end-groups and their sodium salt (+ 22 Da shift), hydroxyl end-groups, as well as methyl esters by considering the original chemical structure of PET and the well-known degradation schemes. However, a few unexpected peaks are also observed in the mass spectrum such as ions at *m/z* 1251.29, 1259.26, and 1281.30. Additional peak assignments were proceeded using the remainder plot analysis [6] derived from the KMD analysis.

**Table 1:**
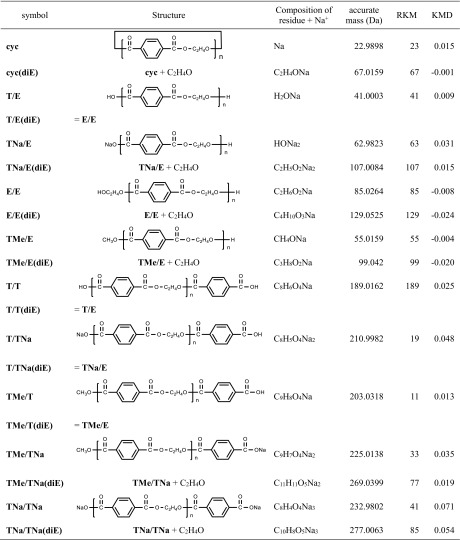
Chemical Structures of the Degradation Products of the PET Samples Observed in Fig. 7 and their Parameters Relating to the RKM Analysis

**Figure 8:**
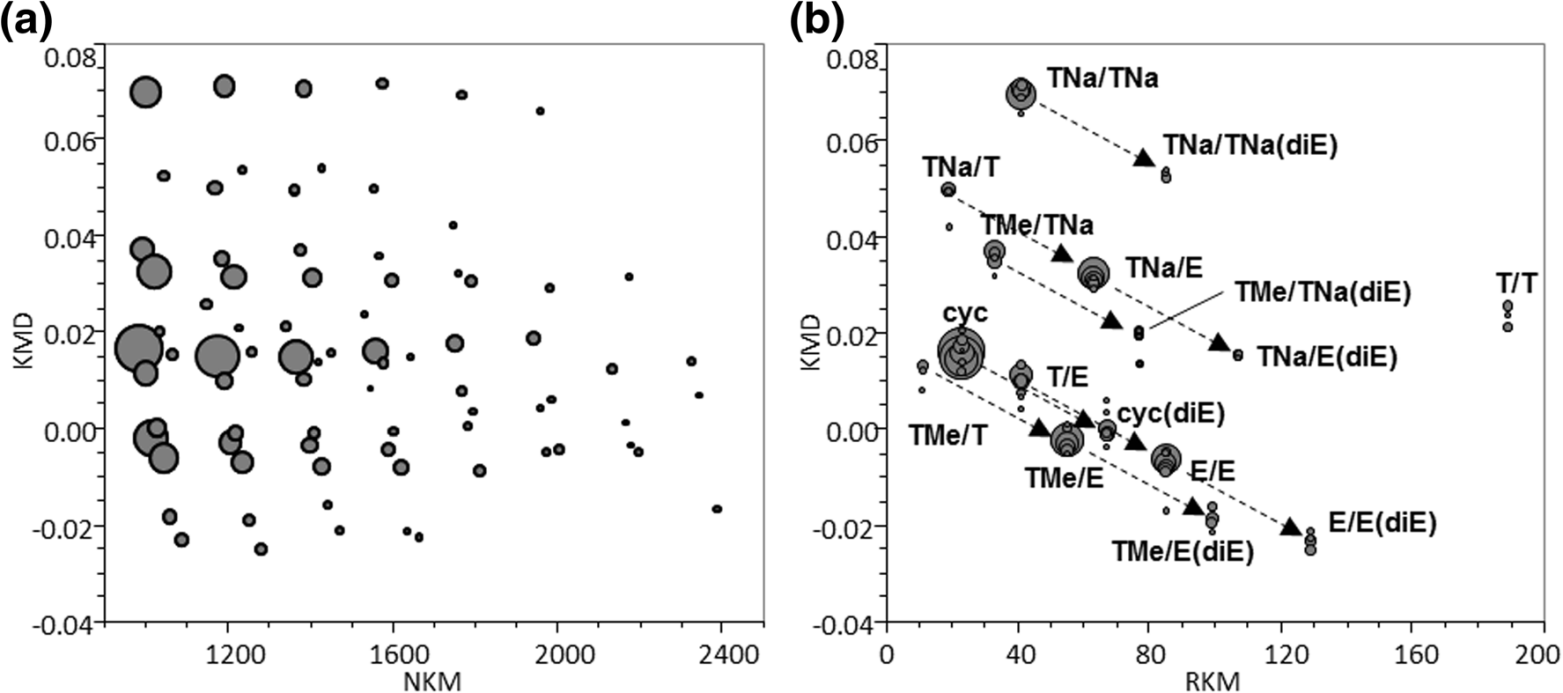
KMD plot (**a**) and remainder plot (**b**) of the degraded PET film sample

Figure 8 displays the results of two types of KMD analysis of the deisotoped mass spectral data. The regular KMD plot (Figure 8a) was computed with *X* = 1 in Eq. (1) and ethylene terephthalate as the base unit (*R* = 192.0423). The other type of KMD plot (Figure8b) is the remainder plot, where the *x*-axis is converted from NKM to the remainder of NKM (RKM) divided by round(*R*) = 192, i.e., RKM = NKM mod 194, which corresponds to the residual mass of the end-groups and the adducted cation (cf. DP plot, previous section). Doing so, the *x*-axis discriminates oligomers by the nature of their end-groups and adducted cation. Ions with the same set of end-groups which line up horizontally in Figure 8a are condensed into a single point with specific coordinates in the remainder plot [6]. For example, the series noted **T/E** (carboxylic acid/hydroxyl terminations) lining up at KMD = 0.009 in the KMD plot and spreading along the *x*-axis is condensed in a single point (RKM, KMD) = (41, 0.009). The point at (85, − 0.008) corresponds to the whole **E/E** series (dihydroxyl-ended PET oligomers), differing from **T/E** by an additional ethylene glycol moiety (C_2_H_4_O, 44 Da). Extending the virtual line which connects these two points in the remainder plot (Figure 8), we reach the point at (129, − 0.024) not yet assigned. Considering that the same interval separates **T/E** and **E/E** as **E/E** and the unknown series, we can speculate that oligomers from this series contain one more ethylene glycol moiety in their backbone compared to **E/E**, consequently assigned as **E/E(diE)** as observed at *m/z* 1281.3068 in Figure 7b, c. Similarly, other unknown compounds at *m/z* 1229.2498, *m/z* 1251.2944, and *m/z* 1259.2610 (Figure 7b, c) are also intuitively found to contain a diethylene glycol moiety using the dot distribution pattern in the remainder plot as a graphical clue (Figure 8b) further assigned to **TMe/TNa(diE)**, **TMe/E(diE)**, and **TNa/E(diE)**, respectively. This assignment is reasonable as the presence of diethylene glycol unit is well known as a by-product of industrial PET [39]. Interestingly, **TNa/TNa(diE)** is also observed in the RKM plot shifted from **TNa/TNa** by one ethylene glycol unit and detected in the mass spectrum at *m/z* 1237.2185. Because both terminus of **TNa/TNa** should be the sodium salt of terephthalate unit, it can be inferred that the diethylene glycol unit exists not only at the chain terminal but also within the main chain. Derived from this assignment, the peak series positioned at (67, − 0.001) in the RKM plot with one oligomer detected at *m/z* ca.1219.27 in all the three mass spectra in Figure 7 can be identified as cyclic oligomers containing one diethylene glycol unit (**cyc(diE)**) rather than the isomeric compound having carboxyl and olefin end-groups produced by β-elimination reaction. This assignment is further supported by the absence of peaks corresponding to the sodium salt and methyl ester of the hypothetic β-elimination products.

## Conclusion

The on-plate degradation procedure based on a partial alkaline transesterification can easily yield oligomeric products from high molecular weight polyesters. This simple and minute-long pretreatment can be performed directly on the MALDI target by pipetting. Since small amounts of sample and reagents are required, isotope labeling is readily done to shift the mass of specific products and track some degradation pathways or avoid isomeric issues. The resulting complex mass spectra with products having different end-group combinations or copolymer compositions—informative but uneasy to decipher manually—were efficiently processed using a series of advanced KMD analysis techniques. A resolution-enhanced KMD plot, a remainder plot, and a DP plot helped at visualizing the molecular weight distribution of oligomers having specific end-group combinations, the distribution of these end-group combinations, and the comonomer distribution. As demonstrated with the characterization of bacterial polyesters and industrial PET samples, the combination of the on-plate degradation pretreatment, high-resolution MALDI TOF MS measurements, and advanced KMD analyses constitutes an effective analytical strategy for the molecular characterization of high molecular weight polyesters.

## Electronic Supplementary Material

ESM 1 (DOCX 184 kb)
