# Supplementary material for: Molecular Characterization of High Molecular Weight Polyesters by Matrix-Assisted Laser Desorption/Ionization High-Resolution Time-of-Flight Mass Spectrometry Combined with On-plate Alkaline Degradation and Mass Defect Analysis
Source: J Am Soc Mass Spectrom. 2018 Nov 8;30(2):355–67. doi: 10.1007/s13361-018-2092-x (PMC6345728; doi:10.1007/s13361-018-2092-x)
Supplement: Supplementary file 1 — (DOCX 184 kb) [file 13361_2018_2092_MOESM1_ESM.docx]

**Supporting Information**

**Molecular characterization of high molecular weight polyesters by matrix-assisted laser desorption/ionization high-resolution time-of-flight mass spectrometry combined with on-plate alkaline degradation and mass defect analysis.**

Sayaka Nakamura^1^, Thierry Fouquet^1^, Hiroaki Sato^1^

^1^ Research Institute for Sustainable Chemistry, National Institute of Advanced Industrial Science and Technology (AIST), Tsukuba, Ibaraki 294-8565, Japan.

**Correspondence to: H. Sato*

*National Institute of Advanced Industrial Science and Technology (AIST)*

*Tsukuba, Ibaraki 294-8565, Japan*

*E-mail: sato-hiroaki@aist.go.jp*

**Supporting information**

Fractionation of poly(ɛ-caprolactone) (PCL) sample:

A high molecular weight fraction of PCL was recovered from a size exclusion chromatography (SEC) elution of the commercial PCL using a HLC8220 system (Tosoh, Tokyo, Japan) equipped with a refractive index detector (RID). 200 μL of the sample solution at 2 mg mL^-1^ in CHCl_3_ were fractionated using two TSKgel multipore HXL-M columns (7.8 mm × 300 mm) connected in series following a multipore Hxl guard column with CHCl_3_ as the mobile phase (1 mL min^-1^). Aliquots of 0.5 mL (from 16 m 30 s – 17 m 0 s) from were collected in vials directly after the RID. The concentration of the chosen fraction could be estimated as 0.08 mg mL^-1^ based on the RID signal intensity. The collected fraction was measured by an AXIMA CFR-plus time-of-flight mass spectrometer (Shimadzu/Kratos, Kyoto, Japan) in positive linear mode. The molecular distribution of the fractionated sample was from *m/z* 10000 to 18000 with the number-average molecular weight (*M*_n_) of about 13800 g mol^-1^. No peak was observed below *m/z* 4000 as shown in Figure 1(a) in the main text.


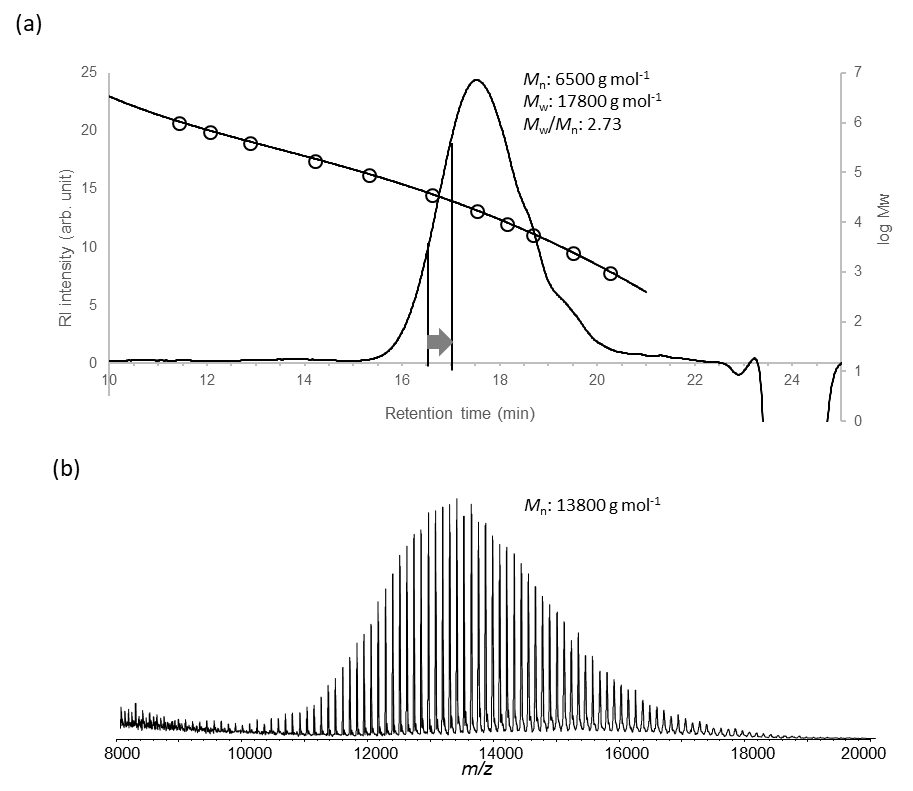


Figure SI-1. SEC chromatogram of PCL with PS calibration curve (a) and the corresponding mass spectrum recorded by AXIMA CFR in linear mode (b).

| 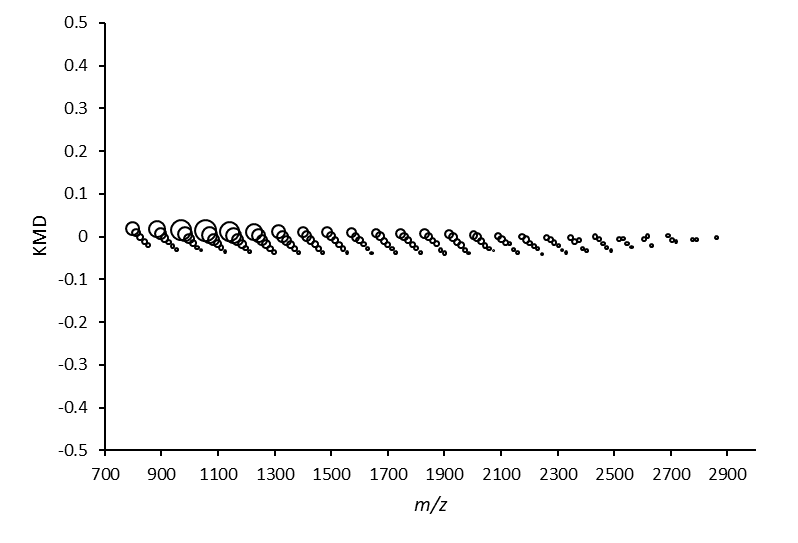  (a) | 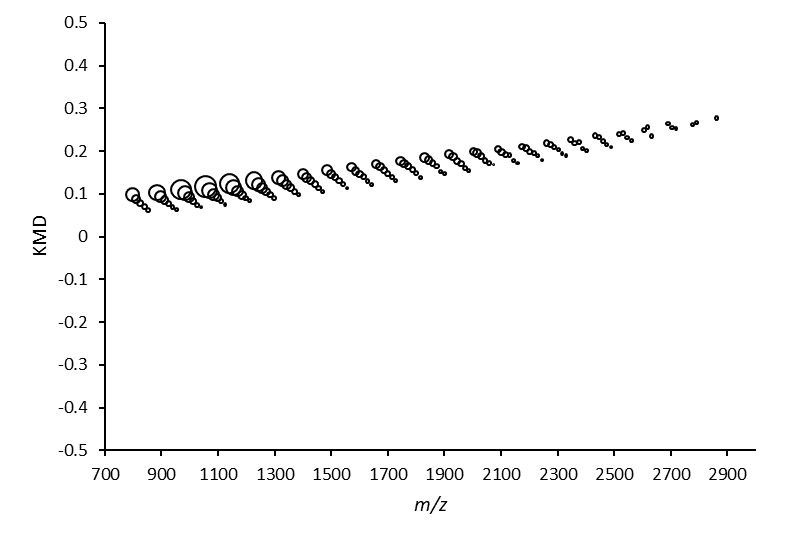  (b) |
| --- | --- |
| 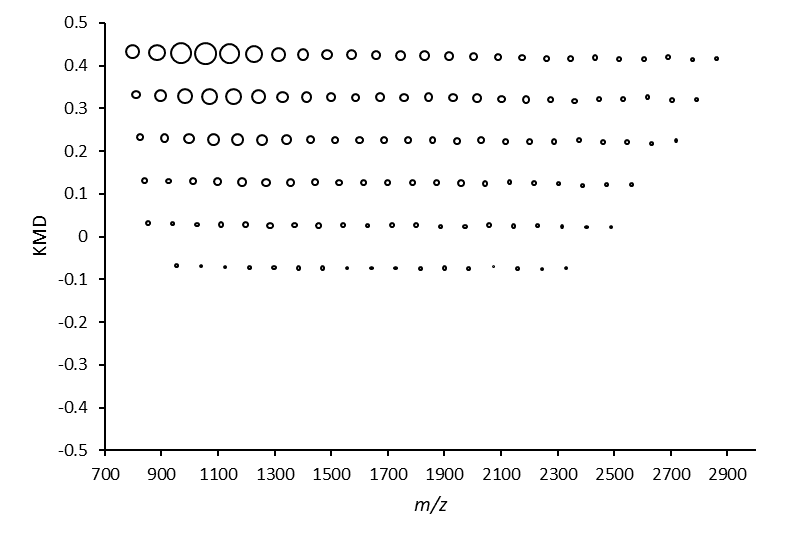  (c) | 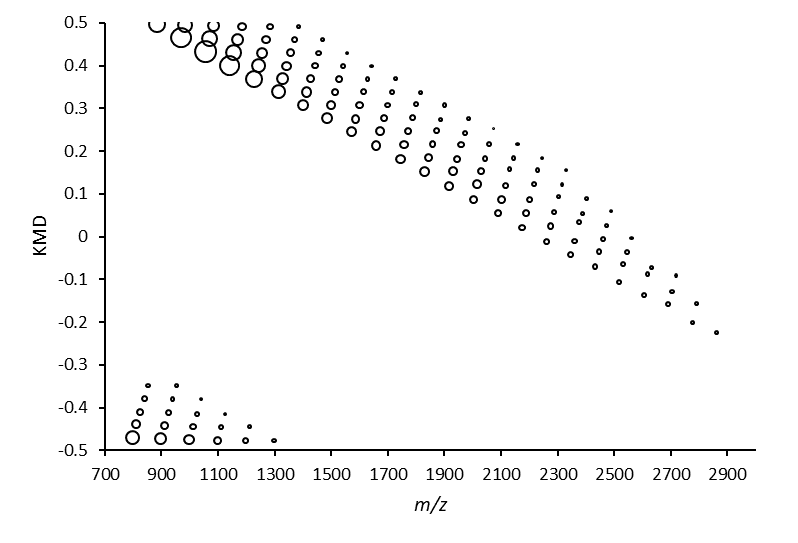  (d) |
| **Figure SI-2.** KMD plots of the type I cloud extracted from the mass spectrum of P(3HB-*co*-3HV) processed by the on-plate degradation using NaOH in methanol-*d*_4_. Regular KMD plots using (a) 3HB (repeat unit: C_4_H_6_O_2_, 86.03623) and (b) 3HV (repeat unit: C_5_H_8_O_2_, 100.0524). Resolution-enhanced KMD plots using (c) 3HB (repeat unit: 86.03623, divisor: X = 62) and (d) 3HV (repeat unit: 100.0524, divisor: X = 114). (e) Resolution-enhanced KMD plot (d) after a -0.2 shift of the points along the y-axis to fit the KMD range with no aliasing. | 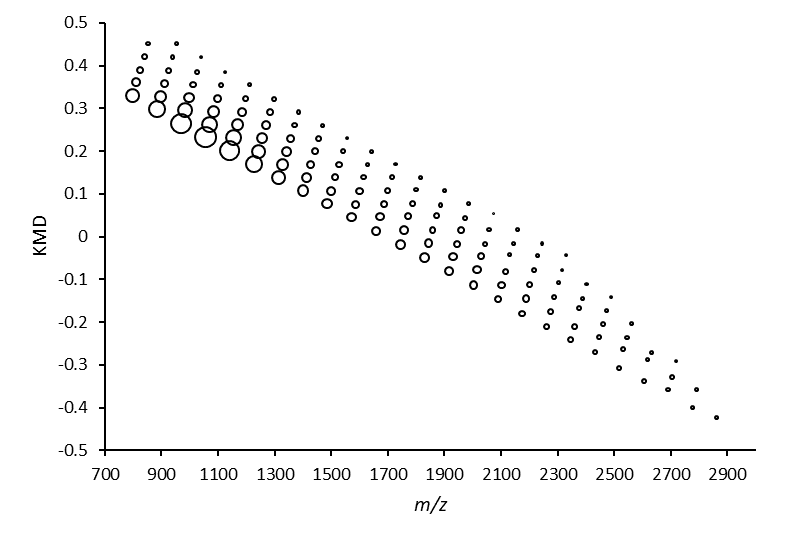  (e) |

As compared to the condensed regular KMD plots (Figure SI-2 (a) and (b)), a careful choice of the divisors X mildly expands the copolymeric distribution over the entire KMD range (from -0.5 to 0.5, Figure SI-2 (c) and (d)). A final shift of the plot along the y-axis prevents from any aliasing (spectral width of a KMD plot: +0.5-(-0.5)=+1, aliased point=point observed at one or several spectral width from its actual position, e.g. KMD_actual_=-0.6, KMD_observed_=-0.6+1=+0.4) (Figure SI-2 (e)).

**Table SI-1.** Mass-to-charge ratios and KMDs of the type I cloud extracted from the mass spectrum of P(3HB-*co*-3HV) processed by the on-plate degradation using NaOH in methanol-*d*_4_ using 3HB (repeat unit: C_4_H_6_O_2_, 86.03623, divisor X=1 and X=62) and 3HV (repeat unit: C_5_H_8_O_2_, 100.0524, divisor X=1 and X=114, -0.2 shift).

| ***m/z*** | **Intensity** | **Regular KMD**  (3HB, X=1) | **Resolution-enhanced**  **KMD** (3HB, X=62) | **Regular KMD**  (3HV, X=1) | **Resolution-enhanced**  **KMD** (3HV, X=114) | **-0.2 shift** |
| --- | --- | --- | --- | --- | --- | --- |
|  |  |  |  |  |  | **KMD(3HV/114)-0.2** |
| 797.316 | 453894 | 0.0198 | 0.4328 | 0.0974 | -0.4689 | 0.3311 |
| 811.332 | 193761 | 0.0097 | 0.3326 | 0.0887 | -0.4389 | 0.3611 |
| 825.349 | 127886 | -0.0010 | 0.2318 | 0.0794 | -0.4095 | 0.3905 |
| 839.364 | 96789 | -0.0106 | 0.1319 | 0.0711 | -0.3789 | 0.4211 |
| 853.380 | 62792 | -0.0203 | 0.0319 | 0.0628 | -0.3484 | 0.4516 |
| 883.355 | 624061 | 0.0173 | 0.4310 | 0.1033 | 0.4978 | 0.2978 |
| 897.371 | 331406 | 0.0072 | 0.3308 | 0.0947 | -0.4721 | 0.3279 |
| 911.387 | 183382 | -0.0029 | 0.2305 | 0.0859 | -0.4421 | 0.3579 |
| 925.402 | 83593 | -0.0126 | 0.1304 | 0.0775 | -0.4116 | 0.3884 |
| 939.417 | 61569 | -0.0218 | 0.0308 | 0.0698 | -0.3805 | 0.4195 |
| 953.431 | 42518 | -0.0296 | -0.0679 | 0.0633 | -0.3479 | 0.4521 |
| 969.393 | 916898 | 0.0154 | 0.4297 | 0.1099 | 0.4653 | 0.2653 |
| 983.409 | 503914 | 0.0056 | 0.3296 | 0.1014 | 0.4956 | 0.2956 |
| 997.425 | 254117 | -0.0047 | 0.2292 | 0.0925 | -0.4745 | 0.3255 |
| 1011.440 | 125757 | -0.0145 | 0.1291 | 0.0840 | -0.4442 | 0.3558 |
| 1025.457 | 64998 | -0.0253 | 0.0283 | 0.0746 | -0.4149 | 0.3851 |
| 1039.469 | 37304 | -0.0313 | -0.0691 | 0.0700 | -0.3802 | 0.4198 |
| 1055.431 | 1018276 | 0.0138 | 0.4285 | 0.1166 | 0.4330 | 0.2330 |
| 1069.447 | 593146 | 0.0038 | 0.3283 | 0.1080 | 0.4631 | 0.2631 |
| 1083.462 | 335834 | -0.0062 | 0.2281 | 0.0994 | 0.4933 | 0.2933 |
| 1097.478 | 171333 | -0.0159 | 0.1281 | 0.0911 | -0.4762 | 0.3238 |
| 1111.494 | 82480 | -0.0255 | 0.0281 | 0.0827 | -0.4457 | 0.3543 |
| 1125.509 | 40338 | -0.0348 | -0.0716 | 0.0749 | -0.4147 | 0.3853 |
| 1141.468 | 879534 | 0.0125 | 0.4276 | 0.1237 | 0.4010 | 0.2010 |
| 1155.484 | 575324 | 0.0025 | 0.3274 | 0.1150 | 0.4312 | 0.2312 |
| 1169.500 | 326926 | -0.0072 | 0.2273 | 0.1067 | 0.4616 | 0.2616 |
| 1183.516 | 188964 | -0.0173 | 0.1270 | 0.0980 | 0.4917 | 0.2917 |
| 1197.531 | 90088 | -0.0264 | 0.0275 | 0.0903 | -0.4771 | 0.3229 |
| 1211.545 | 53400 | -0.0345 | -0.0714 | 0.0835 | -0.4448 | 0.3552 |
| 1227.505 | 661670 | 0.0116 | 0.4270 | 0.1312 | 0.3695 | 0.1695 |
| 1241.521 | 461591 | 0.0015 | 0.3267 | 0.1225 | 0.3996 | 0.1996 |
| 1255.537 | 291264 | -0.0081 | 0.2267 | 0.1142 | 0.4302 | 0.2302 |
| 1269.552 | 175269 | -0.0176 | 0.1269 | 0.1061 | 0.4610 | 0.2610 |
| 1283.568 | 102178 | -0.0274 | 0.0268 | 0.0977 | 0.4914 | 0.2914 |
| 1297.583 | 60607 | -0.0366 | -0.0729 | 0.0898 | -0.4776 | 0.3224 |
| 1313.542 | 460067 | 0.0112 | 0.4267 | 0.1392 | 0.3387 | 0.1387 |
| 1327.558 | 336457 | 0.0012 | 0.3265 | 0.1306 | 0.3688 | 0.1688 |
| 1341.573 | 230543 | -0.0085 | 0.2264 | 0.1222 | 0.3993 | 0.1993 |
| 1355.589 | 155882 | -0.0177 | 0.1267 | 0.1143 | 0.4303 | 0.2303 |
| 1369.604 | 93854 | -0.0272 | 0.0269 | 0.1063 | 0.4612 | 0.2612 |
| 1383.620 | 57723 | -0.0370 | -0.0732 | 0.0978 | 0.4915 | 0.2915 |
| 1399.579 | 315162 | 0.0103 | 0.4260 | 0.1467 | 0.3072 | 0.1072 |
| 1413.594 | 252901 | 0.0012 | 0.3264 | 0.1389 | 0.3384 | 0.1384 |
| 1427.609 | 173244 | -0.0080 | 0.2268 | 0.1311 | 0.3694 | 0.1694 |
| 1441.625 | 123521 | -0.0175 | 0.1269 | 0.1229 | 0.4002 | 0.2002 |
| 1455.641 | 83726 | -0.0279 | 0.0264 | 0.1139 | 0.4298 | 0.2298 |
| 1469.656 | 53048 | -0.0374 | -0.0734 | 0.1058 | 0.4606 | 0.2606 |
| 1485.615 | 258592 | 0.0107 | 0.4263 | 0.1555 | 0.2772 | 0.0772 |
| 1499.631 | 201784 | 0.0010 | 0.3263 | 0.1471 | 0.3077 | 0.1077 |
| 1513.646 | 141072 | -0.0083 | 0.2266 | 0.1392 | 0.3387 | 0.1387 |
| 1527.662 | 105700 | -0.0185 | 0.1262 | 0.1303 | 0.3686 | 0.1686 |
| 1541.677 | 75275 | -0.0278 | 0.0264 | 0.1224 | 0.3995 | 0.1995 |
| 1555.693 | 38707 | -0.0374 | -0.0735 | 0.1142 | 0.4302 | 0.2302 |
| 1571.652 | 231216 | 0.0096 | 0.4255 | 0.1627 | 0.2455 | 0.0455 |
| 1585.669 | 187852 | -0.0008 | 0.3250 | 0.1537 | 0.2752 | 0.0752 |
| 1599.683 | 133613 | -0.0090 | 0.2261 | 0.1469 | 0.3075 | 0.1075 |
| 1613.697 | 94266 | -0.0175 | 0.1269 | 0.1397 | 0.3392 | 0.1392 |
| 1627.714 | 66056 | -0.0282 | 0.0262 | 0.1304 | 0.3686 | 0.1686 |
| 1641.729 | 38563 | -0.0380 | -0.0739 | 0.1219 | 0.3990 | 0.1990 |
| 1657.690 | 220047 | 0.0082 | 0.4245 | 0.1698 | 0.2135 | 0.0135 |
| 1671.703 | 183533 | 0.0010 | 0.3263 | 0.1639 | 0.2468 | 0.0468 |
| 1685.719 | 126787 | -0.0095 | 0.2257 | 0.1547 | 0.2764 | 0.0764 |
| 1699.734 | 90670 | -0.0186 | 0.1261 | 0.1470 | 0.3076 | 0.1076 |
| 1713.750 | 66299 | -0.0279 | 0.0264 | 0.1391 | 0.3385 | 0.1385 |
| 1727.764 | 40093 | -0.0368 | -0.0730 | 0.1316 | 0.3700 | 0.1700 |
| 1743.727 | 231039 | 0.0070 | 0.4236 | 0.1769 | 0.1817 | -0.0183 |
| 1757.740 | 189417 | -0.0001 | 0.3255 | 0.1712 | 0.2151 | 0.0151 |
| 1771.754 | 121988 | -0.0080 | 0.2268 | 0.1646 | 0.2477 | 0.0477 |
| 1785.770 | 96592 | -0.0177 | 0.1268 | 0.1563 | 0.2782 | 0.0782 |
| 1799.784 | 62160 | -0.0263 | 0.0276 | 0.1491 | 0.3099 | 0.1099 |
| 1813.802 | 50970 | -0.0383 | -0.0741 | 0.1384 | 0.3378 | 0.1378 |
| 1829.763 | 237543 | 0.0073 | 0.4239 | 0.1856 | 0.1515 | -0.0485 |
| 1843.776 | 192355 | 0.0000 | 0.3256 | 0.1797 | 0.1848 | -0.0152 |
| 1857.792 | 125528 | -0.0093 | 0.2259 | 0.1717 | 0.2157 | 0.0157 |
| 1871.805 | 92229 | -0.0171 | 0.1272 | 0.1653 | 0.2484 | 0.0484 |
| 1885.825 | 59439 | -0.0312 | 0.0240 | 0.1525 | 0.2739 | 0.0739 |
| 1899.838 | 62593 | -0.0379 | -0.0738 | 0.1472 | 0.3079 | 0.1079 |
| 1915.801 | 197464 | 0.0057 | 0.4227 | 0.1924 | 0.1193 | -0.0807 |
| 1929.814 | 176397 | -0.0012 | 0.3248 | 0.1869 | 0.1530 | -0.0470 |
| 1943.831 | 118708 | -0.0121 | 0.2239 | 0.1773 | 0.1821 | -0.0179 |
| 1957.844 | 116878 | -0.0196 | 0.1254 | 0.1712 | 0.2152 | 0.0152 |
| 1971.862 | 68522 | -0.0316 | 0.0237 | 0.1605 | 0.2429 | 0.0429 |
| 1985.875 | 45428 | -0.0384 | -0.0742 | 0.1551 | 0.2768 | 0.0768 |
| 2001.839 | 176865 | 0.0035 | 0.4211 | 0.1986 | 0.0864 | -0.1136 |
| 2015.850 | 185731 | -0.0012 | 0.3248 | 0.1953 | 0.1226 | -0.0774 |
| 2029.865 | 117057 | -0.0100 | 0.2254 | 0.1878 | 0.1541 | -0.0459 |
| 2043.882 | 89305 | -0.0211 | 0.1243 | 0.1780 | 0.1829 | -0.0171 |
| 2057.894 | 63709 | -0.0279 | 0.0264 | 0.1726 | 0.2168 | 0.0168 |
| 2071.905 | 17626 | -0.0324 | -0.0699 | 0.1694 | 0.2532 | 0.0532 |
| 2087.877 | 130770 | 0.0021 | 0.4201 | 0.2055 | 0.0543 | -0.1457 |
| 2101.891 | 139436 | -0.0062 | 0.3211 | 0.1986 | 0.0864 | -0.1136 |
| 2115.905 | 101445 | -0.0143 | 0.2222 | 0.1918 | 0.1187 | -0.0813 |
| 2129.913 | 64890 | -0.0163 | 0.1278 | 0.1912 | 0.1580 | -0.0420 |
| 2143.933 | 57543 | -0.0302 | 0.0248 | 0.1787 | 0.1837 | -0.0163 |
| 2157.947 | 46311 | -0.0380 | -0.0739 | 0.1723 | 0.2164 | 0.0164 |
| 2173.916 | 106318 | -0.0006 | 0.4182 | 0.2112 | 0.0207 | -0.1793 |
| 2187.928 | 131154 | -0.0069 | 0.3206 | 0.2063 | 0.0552 | -0.1448 |
| 2201.942 | 92214 | -0.0149 | 0.2218 | 0.1996 | 0.0875 | -0.1125 |
| 2215.954 | 73875 | -0.0212 | 0.1243 | 0.1947 | 0.1220 | -0.0780 |
| 2229.967 | 54148 | -0.0281 | 0.0263 | 0.1892 | 0.1557 | -0.0443 |
| 2243.985 | 34476 | -0.0399 | -0.0753 | 0.1787 | 0.1837 | -0.0163 |
| 2259.953 | 100149 | -0.0013 | 0.4176 | 0.2188 | -0.0105 | -0.2105 |
| 2273.964 | 105155 | -0.0067 | 0.3208 | 0.2149 | 0.0250 | -0.1750 |
| 2287.978 | 79588 | -0.0141 | 0.2224 | 0.2088 | 0.0580 | -0.1420 |
| 2301.989 | 52049 | -0.0199 | 0.1252 | 0.2044 | 0.0930 | -0.1070 |
| 2316.006 | 42809 | -0.0311 | 0.0241 | 0.1945 | 0.1217 | -0.0783 |
| 2330.018 | 33329 | -0.0373 | -0.0734 | 0.1897 | 0.1563 | -0.0437 |
| 2345.989 | 101215 | -0.0014 | 0.4176 | 0.2272 | -0.0410 | -0.2410 |
| 2360.005 | 81695 | -0.0112 | 0.3175 | 0.2187 | -0.0107 | -0.2107 |
| 2374.009 | 70330 | -0.0090 | 0.2261 | 0.2223 | 0.0334 | -0.1666 |
| 2388.033 | 54677 | -0.0271 | 0.1200 | 0.2055 | 0.0543 | -0.1457 |
| 2402.045 | 43139 | -0.0333 | 0.0225 | 0.2008 | 0.0889 | -0.1111 |
| 2432.024 | 89597 | -0.0001 | 0.4186 | 0.2369 | -0.0700 | -0.2700 |
| 2446.036 | 80489 | -0.0056 | 0.3215 | 0.2327 | -0.0347 | -0.2347 |
| 2460.052 | 65000 | -0.0160 | 0.2210 | 0.2237 | -0.0050 | -0.2050 |
| 2474.066 | 49687 | -0.0245 | 0.1218 | 0.2165 | 0.0268 | -0.1732 |
| 2488.080 | 39576 | -0.0326 | 0.0230 | 0.2098 | 0.0592 | -0.1408 |
| 2518.066 | 75995 | -0.0055 | 0.4147 | 0.2399 | -0.1066 | -0.3066 |
| 2532.071 | 69020 | -0.0044 | 0.3224 | 0.2423 | -0.0637 | -0.2637 |
| 2546.088 | 65135 | -0.0163 | 0.2208 | 0.2318 | -0.0358 | -0.2358 |
| 2560.102 | 45313 | -0.0242 | 0.1221 | 0.2252 | -0.0033 | -0.2033 |
| 2604.102 | 70633 | -0.0054 | 0.4147 | 0.2484 | -0.1369 | -0.3369 |
| 2618.102 | 61513 | 0.0010 | 0.3263 | 0.2560 | -0.0881 | -0.2881 |
| 2632.130 | 51941 | -0.0213 | 0.2172 | 0.2352 | -0.0719 | -0.2719 |
| 2690.130 | 63637 | 0.0027 | 0.4205 | 0.2648 | -0.1582 | -0.3582 |
| 2704.147 | 60894 | -0.0080 | 0.3198 | 0.2554 | -0.1288 | -0.3288 |
| 2718.156 | 43692 | -0.0113 | 0.2244 | 0.2535 | -0.0910 | -0.2910 |
| 2776.177 | 53203 | -0.0074 | 0.4132 | 0.2630 | -0.2001 | -0.4001 |
| 2790.181 | 50998 | -0.0059 | 0.3214 | 0.2660 | -0.1568 | -0.3568 |
| 2862.207 | 52896 | -0.0014 | 0.4176 | 0.2774 | -0.2237 | -0.4237 |
| 2948.251 | 37805 | -0.0096 | 0.4117 | 0.2776 | -0.2635 | -0.4635 |


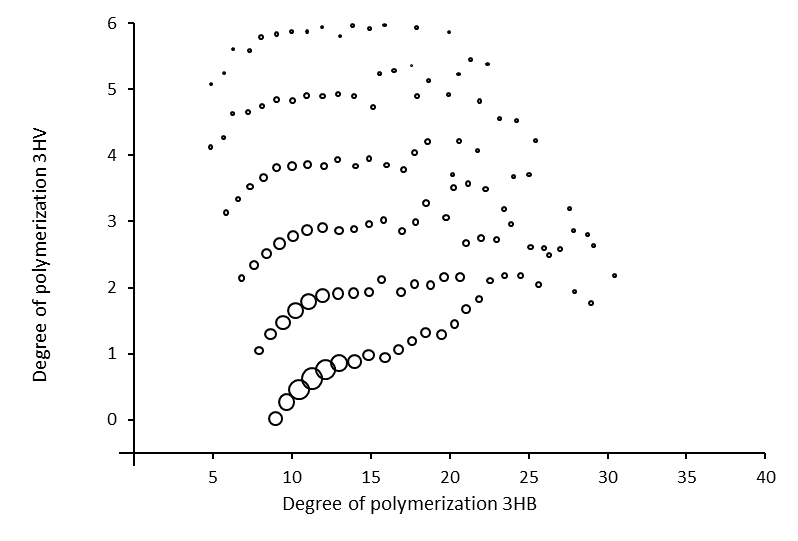

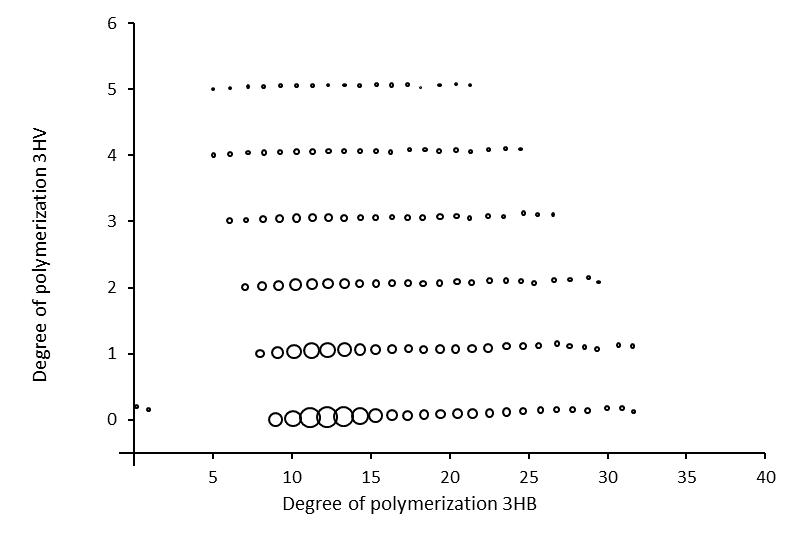


(a)

(b)

**Figure SI-3.** DP plots of the type I cloud extracted from the mass spectrum of P(3HB-co-3HV) processed by the on-plate degradation using NaOH in methanol-*d*_4_. (a) Regular DP plot (repeat units: 3HB C_4_H_6_O_2_, 86.03623; 3HV C_5_H_8_O_2_, 100.0524), (b) resolution-enhanced DP plot (repeat unit: 3HB 86.03623, divisor: X = 62), (c) Regular KMD plot (repeat unit: 3HV C_5_H_8_O_2_, 100.0524, divisor: X=107).

A regular DP plot computed with 3HB and 3HV provides the minimal and maximal degree of polymerization in one and the other co-monomer (x- and y-axis, Figure SI-3 (a)). The limited quality of point alignments in the regular KMD plots nevertheless affect the quality of point alignments in the regular DP plot itself as points are supposed to be positioned at integer values only [S1]. The resolution-enhanced DP plot computed with 3HB/62 and 3HV/114 displays clear horizontal and vertical series of points better emphasizing the discrete composition in 3HB and 3HV. As the divisors have been carefully chosen from the resolution-enhanced KMD plots to avoid strong aliasing (vide supra), no manual anti-aliasing filter with additional parameters (DPmin / DPmax and tolerance) is needed [S1]. A single filter is automatically computed to avoid negative values which requires no manual configuration.

[S1] T. Fouquet, R.B. Cody, H. Sato, Capabilities of the remainders of nominal Kendrick masses and the referenced Kendrick mass defects for copolymer ions, J. Mass Spectrom. 52(9) (2017) 618-624.
